# Identification of a biomarker and immune infiltration in perivascular adipose tissue of abdominal aortic aneurysm

**DOI:** 10.3389/fphys.2022.977910

**Published:** 2022-09-16

**Authors:** Xuming Wang, Bin He, Yisen Deng, Jingwen Liu, Zhaohua Zhang, Weiliang Sun, Yanxiang Gao, Xiaopeng Liu, Yanan Zhen, Zhidong Ye, Peng Liu, Jianyan Wen

**Affiliations:** ^1^ Department of Cardiovascular Surgery, Peking University China-Japan Friendship School of Clinical Medicine, Beijing, China; ^2^ Department of Cardiovascular Surgery, China-Japan Friendship Hospital, Beijing, China; ^3^ Institute of Clinical Medical Sciences, China-Japan Friendship Hospital, Beijing, China; ^4^ Department of Cardiology, China-Japan Friendship Hospital, Beijing, China

**Keywords:** abdominal aortic aneurysm, perivascular adipose tissue, immune infiltration, biomarker, bioinformatics

## Abstract

**Objective:** Abdominal aortic aneurysm (AAA) refers to unusual permanent dilation of the abdominal aorta, and gradual AAA expansion can lead to fatal rupture. However, we lack clear understanding of the pathogenesis of this disease. The effect of perivascular adipose tissue (PVAT) on vascular functional status has attracted increasing attention. Here, we try to identify the potential mechanisms linking AAA and PVAT.

**Methods:** We downloaded dataset GSE119717, including 30 dilated AAA PVAT samples and 30 non-dilated aorta PVAT samples from AAA cases, from Gene Expression Omnibus to identify differentially expressed genes (DEGs). We performed pathway enrichment analysis by Metascape, ClueGo and DAVID to annotate PVAT functional status according to the DEGs. A protein-protein interaction network, the support vector machine (SVM)-recursive feature elimination and the least absolute shrinkage and selection operator regression model were constructed to identify feature genes. Immune infiltration analysis was explored by CIBERSORT. And the correlation between feature gene and immune cells was also calculated. Finally, we used the angiotensin II (Ang II)-ApoE−/− mouse model of AAA to verify the effect of feature gene expression by confirming protein expression using immunohistochemistry and western blot.

**Results:** We identified 22 DEGs, including 21 upregulated genes and 1 downregulated gene. The DEGs were mainly enriched in neutrophil chemotaxis and IL-17 signaling pathway. *FOS* was identified as a good diagnostic feature gene (AUC = 0.964). Immune infiltration analysis showed a higher level of T cells follicular helper, activated NK cells, Monocytes, activated Mast cells in AAA group. And *FOS* was correlated with immune cells. Immunohistochemistry and western blot confirmed higher *FOS* expression in PVAT of the AAA mouse model compared to control group.

**Conclusion:** The differentially expressed genes and pathways identified in this study provide further understanding of how PVAT affects AAA development. FOS was identified as the diagnostic gene. There was an obvious difference in immune cells infiltration between normal and AAA groups.

## Introduction

Abdominal aortic aneurysm (AAA) is caused by pathological dilation of the abdominal aorta, which can lead to rupture from diameter expansion and subsequent death. AAA is one of leading causes of mortality in the elderly (Nordon et al., 2011; Neumann et al., 2019). There are no efficient drugs or potential therapeutic targets for early-stage AAA and no interventions to reverse disease progression, limiting patient care to image surveillance (computed tomography angiography or ultrasound). When the diameter of an AAA expands significantly or causes other complications such as pain, open surgery or endovascular repair are considered to revascularize the aorta (Karthikesalingam et al., 2016). Although there are some biomarkers related to vascular smooth muscle cell proliferation, inflammation, and other immune responses in AAA, they fail to confirm AAA progression sensitively and specifically (Wanhainen et al., 2016). Therefore, finding effective targets to prevent AAA progression should be prioritized.

Perivascular adipose tissue (PVAT) plays a complex role in vascular function. From a protective perspective, PVAT can produce factors such as NO and H2S to attenuate vasocontraction and protect endothelial function (Emilova et al., 2015; Virdis et al., 2015; Xia et al., 2016). On the other hand, PVAT also can promote macrophage infiltration and inflammatory response around the aorta wall. Further, adipose tissue transplantation in the carotid artery can significantly increase atherosclerotic plaque formation (Konaniah et al., 2017). PVAT-derived free fatty acid can activate nuclear factor-κB (NF-κB), reactive oxygen species, and protein kinase C, which may lead to a series of inflammatory downstream activation (Liu et al., 2017; Qi et al., 2018; Rogero and Calder, 2018).

Accumulating evidence demonstrates that PVAT plays an essential role in AAA formation (Kugo et al., 2017). Stimulated by inflammation, PVAT may act as an endocrine tissue, releasing adipokines and maintaining aortic homeostasis (Qi et al., 2018; Piacentini et al., 2020). Macrophages in PVAT could invade the aorta media to augment AAA development, which has been shown in the Ang Ⅱ-induced mouse background (Police et al., 2009). A meta-analysis including 10 animal experiments and 8 human studies showed that AAA is correlated with a high concentration of plasma adipokines. Although several studies have examined the relationship between adipokines and AAA, this relationship remains poorly understood (Thanigaimani and Golledge, 2021). We need conduct more basic experiments to explore the role of PVAT in AAA formation and progression.

Gene expression microarray is a valuable method to explore potential biomarkers and related functions in many diseases (Jie et al., 2020). We used the GSE119717 dataset from Gene Expression Omnibus (GEO) to screen differentially expressed genes (DEGs) between dilated PVAT samples and non-dilated PVAT samples in AAA cases. Further, we identified DEGs that may play a role in AAA and their potential molecular mechanisms, and we verified our discoveries in an animal model of AAA.

## Materials and methods

### Animals

Male ApoE-/- mice (12-week-old, purchased from Beijing HFK Bioscience Co., Ltd.) were fed for one week in a specific-pathogen-free-level lab in the China-Japan Friendship Hospital to improve animal fitness. Mice were randomly assigned to a control group or experimental group. Osmotic mini-pumps (model 2004, Alzet) containing either angiotensin Ⅱ (Ang Ⅱ, 1000 ng/kg/min, Sigma; A9525) or saline were respectively embedded in the experimental group (n = 6) to generate AAA or the control group (n = 6) for 4 weeks. The authors in sequencing paper studied the aneurysmal sac and proximal neck of the same AAA patient in order to avoid confounding effects of other factors, and they excluded Marfan syndrome, recent major surgery (6 months), and auto-inflammatory or immune vascular disease when selecting their patient samples (Piacentini et al., 2019). Similar to their grouping, for the Ang Ⅱ-treated experimental mice, we divided each one into a dilated group (aneurysmal sac, n = 6) and a non-dilated group (proximal neck of AAA, n = 6). Our processing of all mice was based on the principle of random assignment. All operations were performed under aseptic conditions. At 0, 7, 14, 21, 28 days, we measured mouse weight, blood pressure (Tail-cuff system, BP-2000, Visitech Systems, Apex, NC, United States), and aortic diameter (Visual Sonics, Toronto, Canada). After a 28-days standard diet and water, the PVAT of three animals in each group was harvested. Three of each group were frozen in liquid nitrogen, and stored at -80 °C for further western-blot test. And the other 3 in each group were collected and stored in 10% formalin for further histology and immunohistochemistry staining. All procedures complied with the Animal Experimental Ethics Committee of our hospital (Beijing, China) (zryhyy21-21–07–02) and the United States National Institutes of Health Guide for the Care and Use of Laboratory Animals (NIH Publication No. 85–23).

### Microarray dataset and data repeatability test

We downloaded the GSE119717 dataset, based on the GPL10558 platform of HumanHT-12 V4.0 expression beadchip, from GEO (https://www.ncbi.nlm.nih.gov/geo/). This dataset included 30 AAA PVAT samples and 30 non-dilated aorta PVAT samples, obtained from the proximal neck of the aorta from AAA patients. We carried out principal component analysis (PCA) to visualize gene expression and help evaluate sample repeatability and variability. All the data was normalized by “*limma*” package.

### Identification of differentially expressed genes

We used the Bioconductor package “*limma*” in R software to identify significant DEGs between PVAT of AAA samples and control aorta samples in the data. We used *t*-test to calculate *p*-value, with a set cutoff point of adjusted *p* < 0.05, and threshold points of |log fold change| > 1. Additionally, volcano plots and heatmaps were generated in R software to assess DEGs.

### Functional enrichment of differentially expressed genes

Gene ontology (GO)—which consists of three essential aspects: biological process, molecular function, and cellular component—and Kyoto Encyclopedia of Genes and Genomes (KEGG) analysis were conducted to assess the biological mechanisms associated with DEGs. KEGG is an integrated database used to understand advanced biological functions and protein interaction networks at the molecular level. We used DAVID (Sherman et al., 2022), Metascape (Zhou et al., 2019) and ClueGO (Bindea et al., 2009) to perform pathway enrichment. These three tools have different algorithms which could play a role of mutual verification. *p* < 0.05 was considered as statistically significant.

Construction and Analysis of Protein-Protein Interaction Network and Identification of Hub Genes.

To identify the potential protein-protein interaction (PPI) network according to the identified DEGs, we used the Search Tool for the Retrieval of Interacting Genes (STRING) database. STRING is an online tool that provides direct and indirect correlations of PPI (Szklarczyk et al., 2015). We used the Cytoscape software cytoHubba plug-in to clarify hub genes, visualize the PPI network, and select the top 5 hub genes with the greatest degree rank.

### Feature genes selection

We used the least absolute shrinkage and selection operator (LASSO) logistic regression (Tibshirani, 1996) and support vector machine-recursive feature elimination (SVM-RFE) (Guyon et al., 2002) to select the potential feature genes. The LASSO algorithm was based on penalty coefficient by “*glmnet*” R package. And the SVM-RFE was a machine learning technique conducted by “*e1071*” R package to select the optimal variables. We integrated the genes obtained from PPI network, LASSO and SVM-RFE to select the most significant feature genes by Venn plot (Bardou et al., 2014).

### Immune cells infiltration and correlation analysis between feature genes and immune cell

CIBERSORT (https://cibersortx.stanford.edu/) was used to acquire immune cells infiltration matrix with *p* < 0.05 as a result of our submitting normalized GSE119717 gene expression data. The “*vioplot*” package was used to visualize the different immune cells in two groups. And the bar plot was conducted to show the percentage of each immune cell. A correlation heatmap of 22 immune cells was constructed by “*corrplot*” package. The relationship between feature genes and immune cells was analyzed by Spearman’s rank correlation test and the plot was visualized by “*ggplot2*” package.

### Histology and immunohistochemistry

Formalin-embedded aorta samples were sliced into 4-μm sections and underwent sequential dewaxing and rehydration. Sections were stained with hematoxylin and eosin (H-E), Masson’s staining, and elastin van Gieson (kit from Shanghai Yuanmu Biological Technology Co., Ltd. R0490). For immunohistochemistry, sections were blocked and incubated with primary antibodies to FOS (1:250, Proteintech; 66590-1-lg) for 2 h. The target gene was stained yellow-brown, and the total tissue area and integrated optic density (IOD) were measured using Image-Pro Plus 6.0 software (IPP 6.0, Media Cybernetics, United States). IOD per unit area represented the level of gene expression.

### Western blot analysis

We extracted PVAT sample protein using the Solarbio protein extraction kit (BC3710). A BCA protein assay kit (LABLEAD, B5000-500T) was used to determine protein concentration. Equal amounts of PVAT protein were separated by 10% SDS-PAGE and transferred to polyvinylidene fluoride membranes. After blocking with 5% nonfat milk for 1 h, we incubated membranes with primary antibodies to FOS (1:5000, Proteintech; 66590-1-lg) overnight at 4°C. Membranes then were incubated with HRP-conjugated secondary antibody for 1 h at room temperature. We used PierceTM ECL Western Blotting Substrate (Thermo Scientific; Cat: 32209) to detect immunoreactive bands. Bio‐Rad ChemiDoc XRS image analysis system (Bio‐Rad) was used to perform quantitative analysis according to band density.

### Statistical analysis

All collected data were analyzed by R software and GraphPad Prism 8 and are represented as mean ± standard deviation. Differences among normally distributed values of two experimental groups were analyzed by Student’s t-test. Differences of one parameter between normally distributed values of three or more experimental groups were determined by one-way ANOVA. Statistical significance was defined as *p* < 0.05.

## Results

### Dataset validation and identification of differentially expressed genes

**FIGURE 1:**
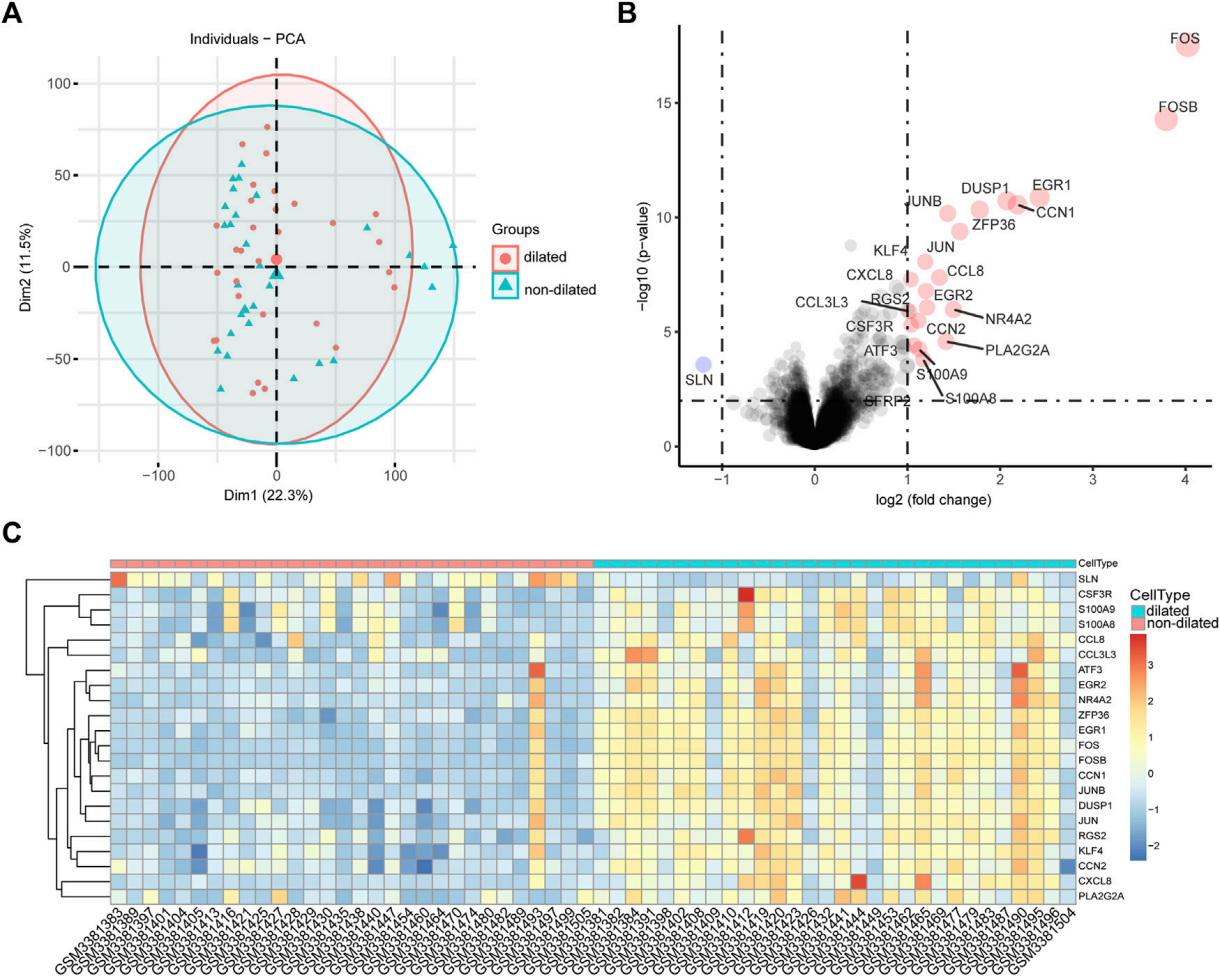
Intra-group repeatability for GSE119717 dataset. **(A)** All samples from the GSE119717 dataset were analyzed by principal component analysis. Principal component 1 is on the *X*-axis; principal component 2 is on the *Y*-axis. **(B)** Volcano plot showing DEGs between the two groups. *X*-axis represents the |log fold change|; *Y*-axis represents the *p*-value (log-scaled). The red dots represent the up-regulated DEGs and the blue dot represents the down-regulated DEGs. **(C)** Heatmap visualizing DEGs between the two groups.

PCA were used to validate GSE119717 dataset quality and to visualize associations between dilated and non-dilated PVAT (Figure 1A). The distance between dilated and non-dilated group was close because these samples in two groups were obtained from different parts of the same individual. We identified 22 DEGs in total, including 1 downregulated and 21 upregulated genes according to the cut-off point. The volcano plot and heatmap were used to show the distribution and expression of DEGs (Figures 1B,C).

### Functional and pathway enrichment

**FIGURE 2:**
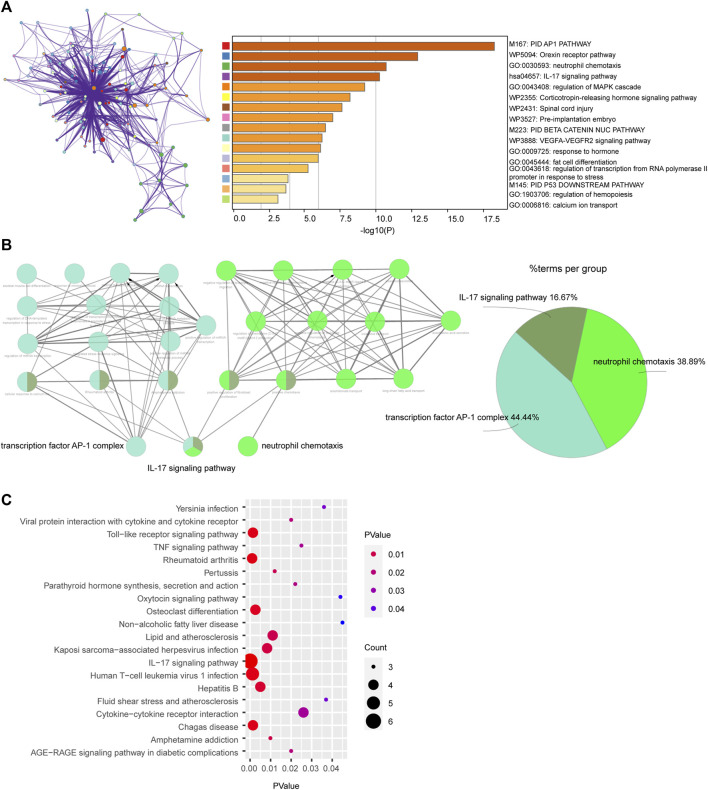
Enrichment of differentially expressed genes (DEGs) via **(A)** Metascape, **(B)** ClueGo and **(C)** DAVID.

First, we uploaded the DEGs to Metascape, the result indicated that these genes mainly enriched were involved in PID AP1 pathway, Orexin receptor pathway, neutrophil chemotaxis and IL-17 signaling pathway (Figure 2A). Second, 22 DEGs were uploaded to ClueGo in ctyoscape to analyze biological process and KEGG. The result showed that transcription factor AP-1 complex, IL-17 signaling pathway and neutrophil chemotaxis were significantly enriched. And these three relative pathways account for 44.44%, 16.67% and 38.89%, respectively (Figure 2B). And the pathway enrichment analysis in DAVID revealed that the DEGs were remarkably enriched in IL-17 signaling pathway, Human T−cell leukemia virus 1 infection, Toll−like receptor signaling pathway and Rheumatoid arthritis (Figure 2C). Notably, IL-17 signaling pathway was the most important pathway in Metascape, ClueGo and DAVID. And neutrophil chemotaxis was enriched by Metascape and ClueGo.

### Feature genes and biomarkers screening

**FIGURE 3:**
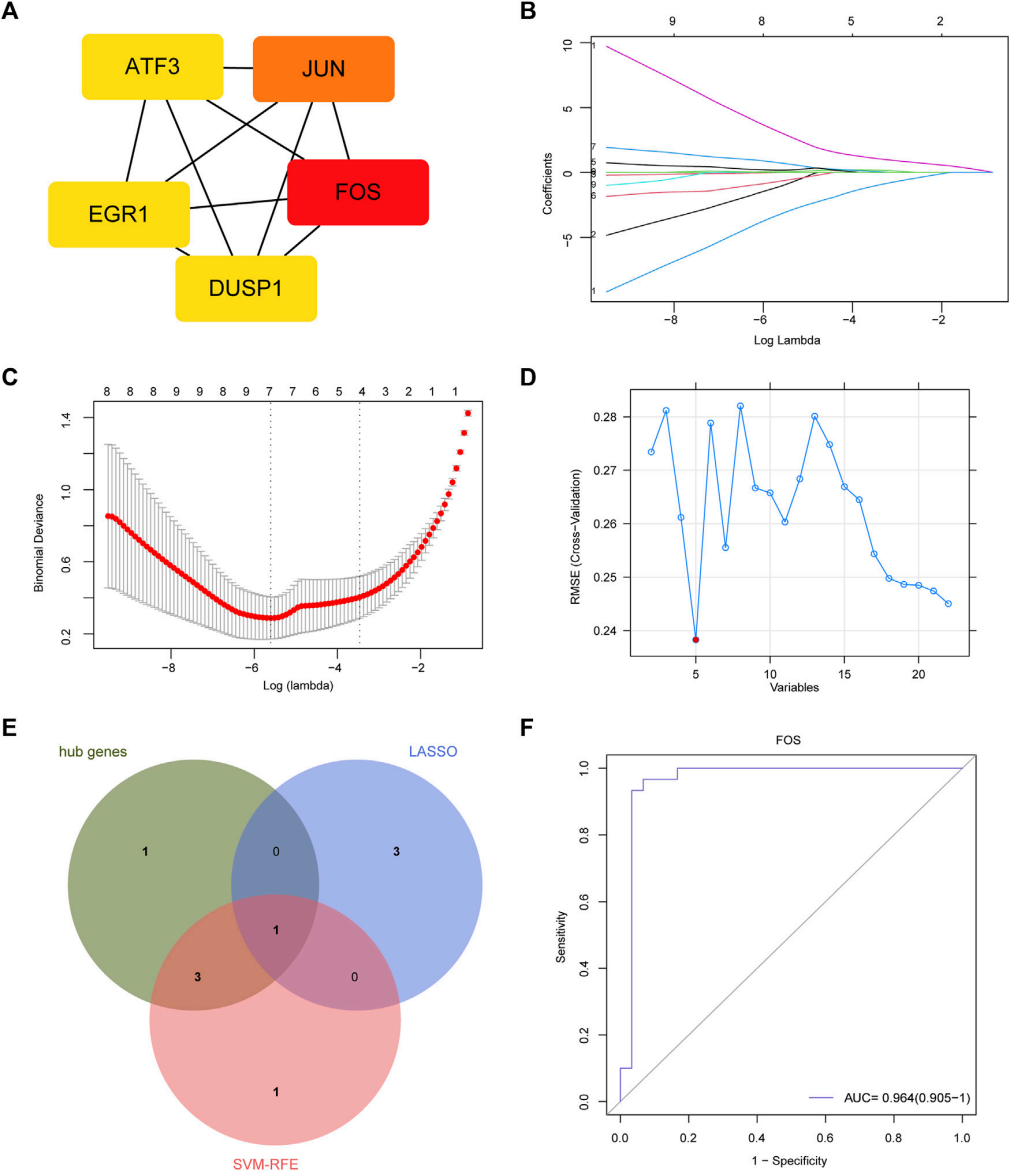
Feature gene and its diagnostic value. **(A)** Protein-protein interaction (PPI) network and hub genes: *FOS, JUN, ATF3, DUSP1, EGR1*. **(B)** LASSO coefficient profiles. **(C)** Identification of the optimal penalization coefficient (lambda) in the Lasso regression and the minimum absolute contraction criterion. **(D)** A plot of feature genes selected by SVM-RFE machine learning. The red dot represents the best five variables. **(E)** Venn plot demonstrates *FOS* was an important marker combined by LASSO, SVM-RFE and hub genes. **(F)** The diagnostic performance of *FOS* according to its expression. AUC, area under the ROC curve.

We used Cytoscape software to show the PPI network, which was constructed according to the STRING database. Using the cytoHubba method to identify genes with the greatest degree rank, the top 5 hub genes were: *FOS, JUN, ATF3, DUSP1, EGR1* (Figure 3A). All the DEGs were used to conduct LASSO regression and SVM-RFE machine learning. The LASSO logistic regression analysis was used to obtain 4 genes: *CSF3R, CXCL8, FOS, SLN* (Figures 3B,C). And the minimum absolute contraction criterion was 0.003678. The SVM-RFE identified 5 genes: *FOS, FOSB, EGR1, DUSP, JUN* (Figure 3D). By combining the genes obtained from these three methods, *FOS* was thought as the most important feature gene in PVAT of AAA (Figure 3E). And we tested the diagnostic efficacy of *FOS*, a high level of area under the curve (0.964) showed that *FOS* had a good diagnostic value (Figure 3F).

### Immune infiltration and correlation between immune cells and FOS

**FIGURE 4:**
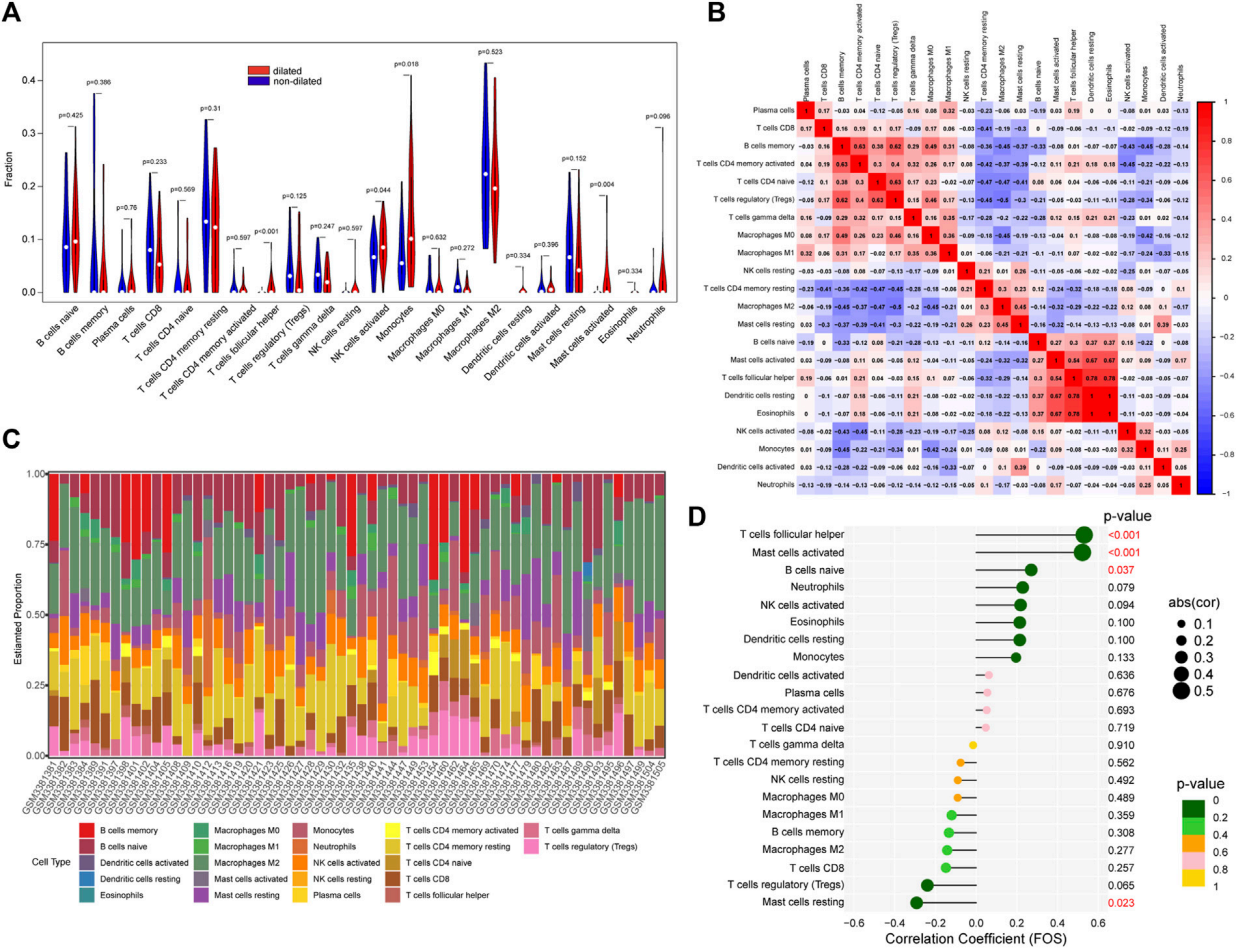
The immune cells infiltration between dilated and non-dilated groups. **(A)** The difference between dilated and non-dilated PVAT groups. Red area represents dilated group and blue area represents non-dilated group. *p* < 0.05 was considered as significant. **(B)** Correlation heatmap of immune cells. Red represents positive correlation and blue represents negative correlation. **(C)** The percentage of each immune cell in each sample. **(D)** The correlation between *FOS* and immune cells.

IL-17 played an important role in regulating immune response (Ruiz de Morales et al., 2020). So, we conduct immune cells infiltration analysis by CIBERSORT. The vioplot indicated that PVAT in expanded aorta area had higher level of follicular helper T cells, activated NK cells, Monocytes, activated Mast cells (Figure 4A). As showed in Figure 4B, the correlation of immune cells indicated that activated CD4 memory T cells were positively correlated with B cells memory (r = 0.63). Regulatory T cells were positively correlated with memory B cells (r = 0.62) and CD4 naïve T cells (r = 0.63), but negatively correlated with M2 macrophages (r = -0.5). Activated Mast cells were positively correlated with follicular helper T cells (r = 0.54), resting Dendritic cells (r = 0.67), Eosinophils (r = 0.67). And follicular helper T cells were positively correlated with resting Dendritic cells (r = 0.78) and Eosinophils (r = 0.78). The percentage of each immune cell was showed in Figure 4C. We further investigated the relationship between FOS and immune cells (Figure 4D). *FOS* was positively correlated with follicular helper T cells (r = 0.53, *p*<0.001), activated Mast cells (r = 0.52, *p* < 0.001) and naïve B cells (r = 0.27, *p* = 0.037), but negatively correlated with resting Mast cells (r = -0.29, *p* = 0.023).

### Construction of abdominal aortic aneurysm animal model and FOS expression

**FIGURE 5:**
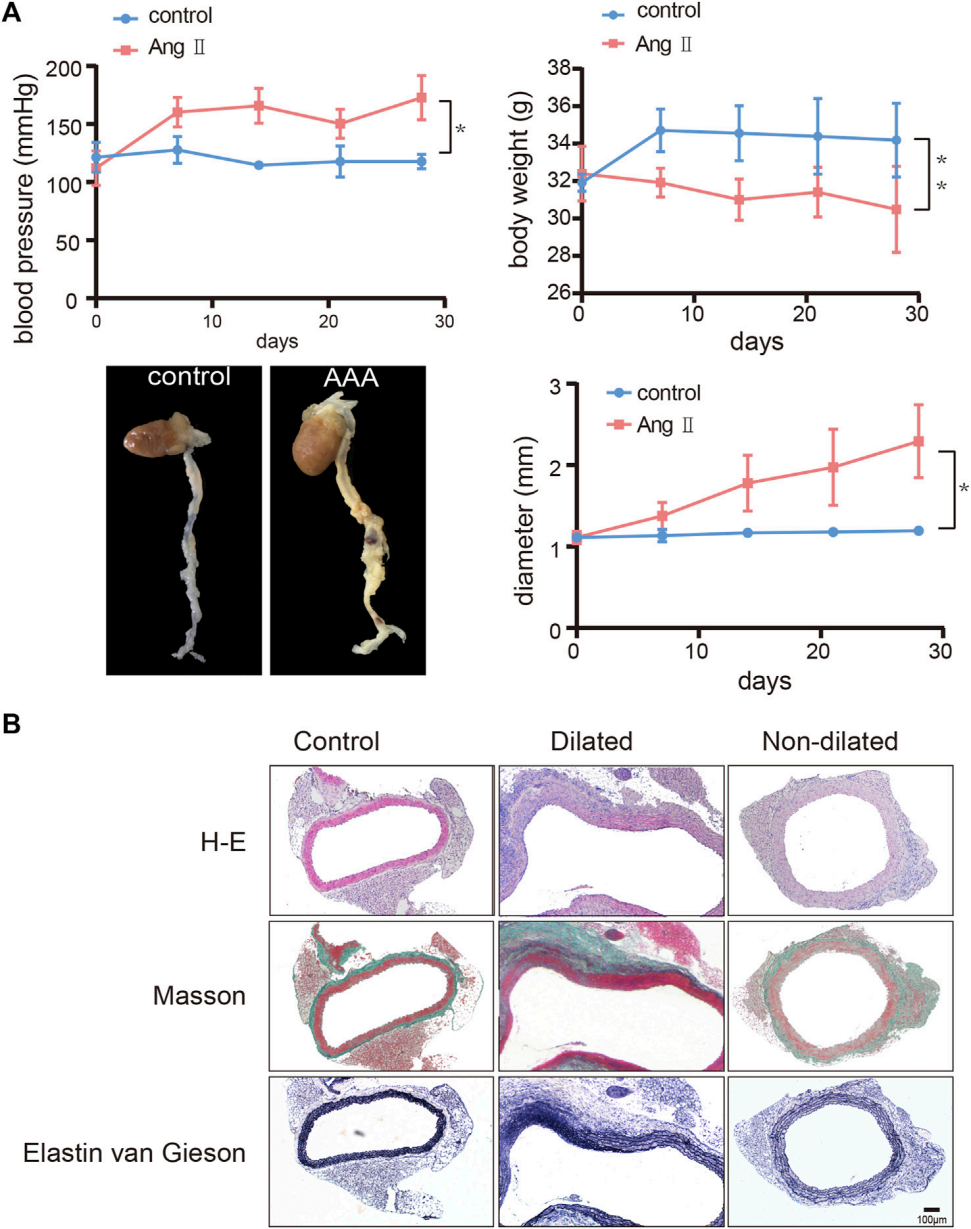
Construction of the abdominal aortic aneurysm (AAA) mouse model. **(A)** Change of blood pressure, body weight, and diameter of the abdominal aorta over 28 days and representative images of harvested hearts and vessels from control and AAA mice. N = 6, results are expressed as the mean ± SD. *p < 0.05, **p < 0.01. Data were analyzed using student t-test. Representative images of **(B)** H-E staining, Masson’s staining, and van Gieson staining of the abdominal aorta from control and AAA mice. Scale bar = 100 μm.

**FIGURE 6:**
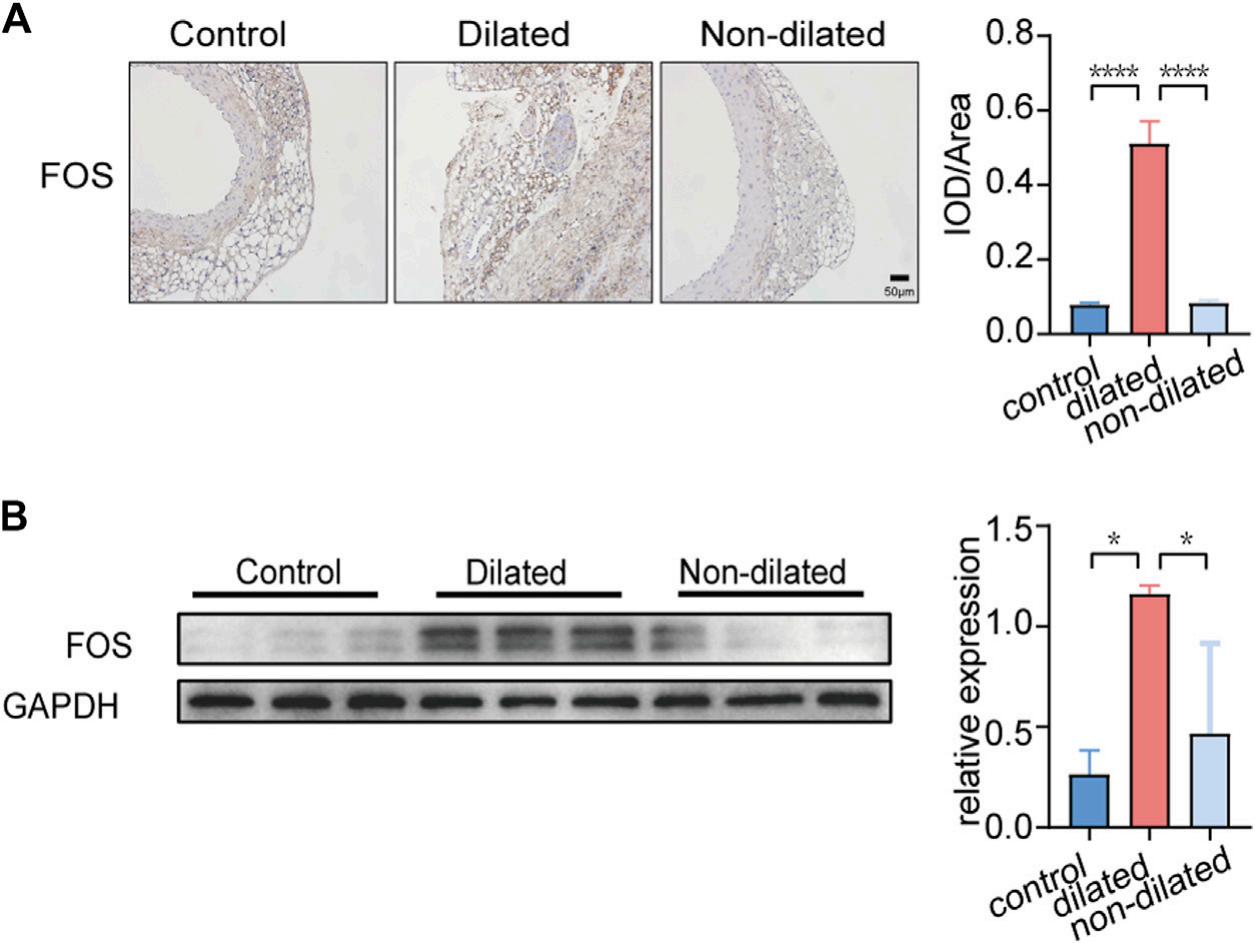
FOS expression in PVAT of control and AAA mice. **(A)** Immunohistochemistry staining of FOS expression. Scale bar = 50μm. *p < 0.05. Data were analyzed by one-way ANOVA. **(B)** Western blot analysis and corresponding quantification of FOS expression (GAPDH is loading control), *p < 0.05. Data were analyzed by one-way ANOVA.

We used Ang Ⅱ (1000 ng/kg/min) to build an AAA model in the ApoE-/- mouse. The main difference between dilated and non-dilated abdominal aorta within the same individual is that the elasticity and compliance of normal vessel wall are lost in the dilated group, causing the wall to become distorted and dilated (Derwich et al., 2020). In our research, we measured mouse weight, blood pressure, and aortic diameter to track AAA progression every 7 days. Although no mice died during our 4-week feeding period, the aneurysm in the AAA group had shown considerable enlargement in diameter and complete rupture of the elastic fiber layer encasing the organ cavity as we showed in Figure 5B and Supplementary Figure S1, forming a pseudoaneurysm in a very high-risk state. Compared with the saline controls, Ang Ⅱ-induced mice presented significantly higher blood pressure, lower body weight, and dilated abdominal aortas (Figure 5A). In addition, H-E staining suggested obvious distortion and expansion of the vascular cavity, Masson’s trichrome staining showed collagen hyperplasia, and van Gieson staining revealed broken elastic fibers in the AAA model (Figure 5B and Supplementary Figure S1). We also verified the diagnostic value of *FOS* in our AAA model, immunohistochemistry (Figure 6A and Supplementary Figure S2) and western blot (Figure 6B) showed increased expression of *FOS* in the AAA model compared to control mice and non-dilated area. These results showed that *FOS* is a reliable diagnostic biomarker to discriminate AAA from normal samples.

## Discussion

The function of PVAT in AAA formation has drawn more attention recently. Inflammatory cells produced by a dysfunctional PVAT infiltrate the abdominal aortic vessel wall and promote inflammation (Omar et al., 2014; Chen et al., 2020). PVAT promotes the expression of MMP-2 and MMP-9, which disrupts collagen and elastic fibers in the abdominal aortic wall, making the wall more prone to dilation and fracture (Kurobe et al., 2013; Horimatsu et al., 2017). Additionally, stem cells and immune cells, particularly T cells, produced from PVAT have a substantial correlation with AAA size (Ye et al., 2021). However, the specific mechanism of PVAT-derived genes and factors with AAA remains unclear, so we performed a bioinformatics analysis of the GSE119717 dataset expecting in the hopes of uncovering new points for the unique action.

Our bioinformatics analysis found 22 DEGs, 21 upregulated and 1 downregulated, in PVAT from AAA. IL-17 signaling pathway and neutrophil chemotaxis were two major pathways we enriched in this analysis. It is generally recognized that AAA is caused by inflammation that destroys the arterial wall and causes abnormal expansion of the aorta (Shimizu et al., 2006). IL-17 has already been found elevated in human inflammatory or immune diseases (Albanesi et al., 1999; Ma et al., 1999). Produced by adaptive and innate immune cells including CD4^+^ Th17 cells, CD8^+^ Tc17 cells, IL-17 predominantly acts to regulate and amplify signals during interaction with other cytokines such as TNF (Majumder and McGeachy, 2021). TNF-α is considered to promote inflammation, and TNF-α expression is upregulated in adipose-derived mesenchymal stem cells of AAA patients (Huang et al., 2019). Inhibiting TNF-α expression can reduce matrix destruction and inflammatory infiltration as well as attenuate AAA pathogenesis, and TNF-α plays an important role in matrix remodeling (Xiong et al., 2009; Wang et al., 2019). Moreover, it has been reported that smooth muscle cell derived IL-17C could recruit TH17 cells to perivascular and promotes atherosclerosis (Galkina et al., 2006). IL-17 can also stimulate neutrophil chemotaxis by inducing the expression of proinflammatory cytokines (Strzępa and Szczepanik, 2011).

It was found that activated T cells are highly aggregated in the PVAT around the AAA and that they can produce inflammatory substances to migrate to the abdominal aortic vessel wall, which is closely related to the size of the AAA (Xiong et al., 2004; Sagan et al., 2019). T cells in PVAT can produce IL17, which in turn promotes the expression of downstream inflammatory factors and MMP (Nosalski and Guzik, 2017; Berry et al., 2022). Similarly, IL17 has been found to be pathogenic in AAA. IL17 are significantly increased in AAA patients and animal models and promote smooth muscle cell remodeling (Sharma et al., 2012). However, there are no experiments to conclusively validate PVAT-IL17-AAA as a pathogenic mechanism in the formation of AAA. Given the strong relationship among the three, we believe that this is a promising pathway for further study.

Many immune cells participate the formation of AAA (Spear et al., 2015). Those immune cells are also observed in PVAT and increase the probability of AAA formation (Police et al., 2009). Due to the close relationship between IL-17 and immune cells, we further investigated immune cell in PVAT by CIBERSORT. Our immune cells infiltration showed that there was significantly difference between PVAT from AAA and control group. T cells in PVAT could express CD25, CD44 and CD69 markers and express humorous receptors for inflammatory cytokines (Antony et al., 2010). The cytokines produced by immune cells in PVAT could regulate the proliferation and migration of vascular smooth muscle cells (McMaster et al., 2015). IL-17A could induce the deposition of collagen and change the compliance of aorta. Knockout of IL-17A could reduce superoxide production and fibrosis. And TNF-α and IL-17A act synergistically to increase human aortic smooth muscle cell expression of CCL8, CSF3, CXCL2, and CCL7 (Madhur et al., 2010). And the cytokines produced by immune cells can also affect on perivascular adipocytes. It has been proved that leptin has pro-inflammatory effects and adiponectin has anti-inflammatory effects. IL-17A and TNF-α could inhibit the production of adiponectin whereas promote the production of leptin (Nosalski and Guzik, 2017).

*FOS* was identified as the most valuable diagnostic biomarker by combining LASSO regression, SVM-RFE machine learning and hub genes. In this study, *FOS* was upregulated in PVAT of AAA and positively correlated with follicular helper T cells, activated Mast cells and naïve B cells whereas negatively correlated with resting Mast cells, which indicates that *FOS* may participate the activation of immune and inflammatory response and play a pro-inflammatory role. *FOS* and *JUN* constitute activator protein-1 (AP-1), which can be induced by TNF-α in inflammation. Activation of AP-1 can result in the membrane attack complex inducing higher expression of MMP2 and MMP9, which are the main proteinases in AAA (Wu et al., 2010). Additionally, increased levels of *FOS* have been shown in aortic aneurysm (Wu et al., 2010). After 4 weeks, the Ang Ⅱ induced AAA model showed significantly aorta expansion and elastic fibers rupture. We conducted immunohistochemistry staining and western-blot to explore the expression of *FOS* in PVAT. Our results support the bioinformatic result that expression of *FOS* increased in the PVAT of the AAA model. Yet this elevation remains controversial. Previous work has shown that miR-155-5p which could inhibit the viability of vascular smooth muscle cells was upregulated in human AAA samples and the expression of *FOS* was significantly less than normal. Increasing *FOS* expression could weaken the effect of miR-155-5p mimic on vascular smooth muscle cells viability (Zhao et al., 2019). It seems that FOS acts as a double-edged sword in regulating vascular smooth muscle cells. More experiments are needed to validated the role of *FOS*.

There are several limitations to our research. First, we use the Ang Ⅱ-induced AAA mouse model for verification instead of human samples and we did not compare standard mice with ApoE mice. Second, the feature gene we verified may be altered during sample harvesting and preservation. Third, the immune infiltration is inferred by bioinformatic method which may have inevitable limitations and further experiments should be carried out to validate this view. Additionally, there is a substantial relationship between aortic PVAT density and aneurysm diameter in the same individual (Dias-Neto et al., 2018). Our investigation, however, was unable to adequately analyze the density of PVAT among various mice and the link between PVAT quantity and AAA development. Further improvement could be achieved by expanding the sample size and performing CT examinations to compare the inflammatory expression in PVAT in different AAA groups.

## Conclusion

We identified 22 DEGs, and pathway enrichment analysis showed different inflammatory signaling pathways were affected in PVAT of AAA compared to non-dilated aorta tissue. Immune cells have a vital role in PVAT during the formation of AAA. *FOS* was identified as the diagnostic biomarker that likely participate in the PVAT inflammatory response to promote AAA formation.

## Data Availability

Publicly available datasets were analyzed in this study. This data can be found here: https://www.ncbi.nlm.nih.gov/geo/; GSE119717.
