# Supplementary figures and images for: Identification of a biomarker and immune infiltration in perivascular adipose tissue of abdominal aortic aneurysm
Source: Front Physiol. 2022 Sep 16;13:977910. doi: 10.3389/fphys.2022.977910 (PMC9523244; doi:10.3389/fphys.2022.977910)

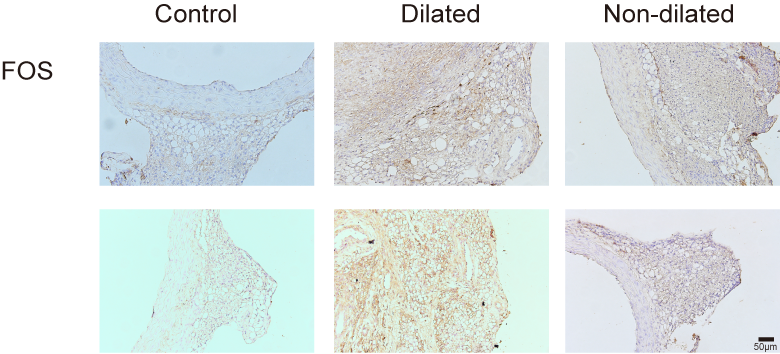

Supplement: Supplementary file 1 [file Image2.TIF]

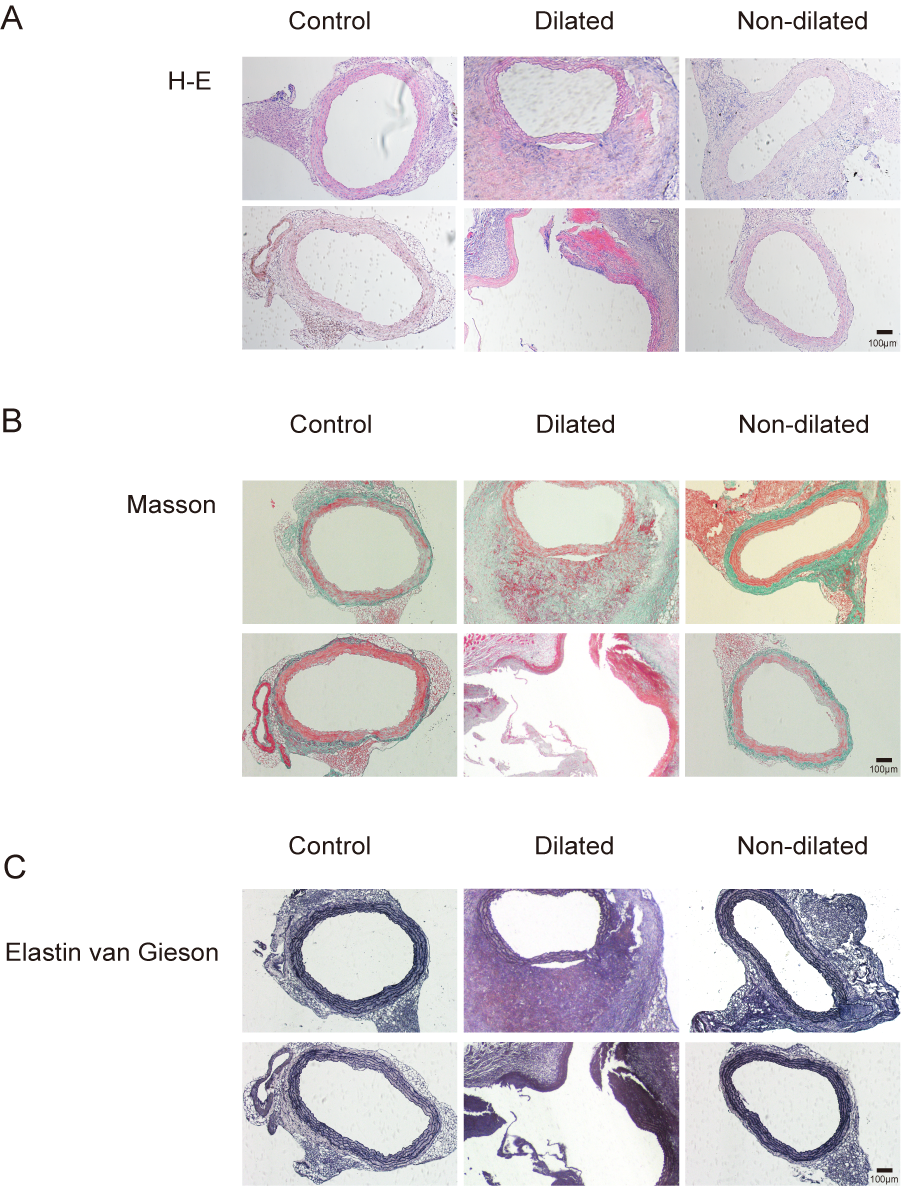

Supplement: Supplementary file 2 [file Image1.TIF]
